# The Precision of Place Fields Governs Their Fate across Epochs of Experience

**DOI:** 10.1523/ENEURO.0261-23.2023

**Published:** 2023-12-06

**Authors:** YuHung Chiu, Can Dong, Seetha Krishnan, Mark E. J. Sheffield

**Affiliations:** 1 Department of Physics, University of Chicago, Chicago, 60637, IL; 2 Department of Neurobiology, University of Chicago, Chicago, 60637, IL; 3 Institute for Neuroscience, University of Chicago, Chicago, 60637, IL

**Keywords:** drift, hippocampus, memory, place cells, spatial representations

## Abstract

Spatial memories are represented by hippocampal place cells during navigation. This spatial code is dynamic, undergoing changes across time, known as representational drift, and across changes in internal state, even while navigating the same spatial environment with consistent behavior. A dynamic code may provide the hippocampus a means to track distinct epochs of experience that occur at different times or during different internal states and update spatial memories. Changes to the spatial code include place fields (PFs) that remap to new locations and place fields that vanish, while others are stable. However, what determines place field fate across epochs remains unclear. We measured the lap-by-lap properties of place cells in mice during navigation for a block of trials in a rewarded virtual environment. We then determined the position of the place fields in another block of trials in the same spatial environment either separated by a day (a distinct temporal epoch) or during the same session but with reward removed to change reward expectation (a distinct internal state epoch). We found that place cells with remapped place fields across epochs tended to have lower spatial precision during navigation in the initial epoch. Place cells with stable or vanished place fields tended to have higher spatial precision. We conclude that place cells with less precise place fields have greater spatial flexibility, allowing them to respond to, and track, distinct epochs of experience in the same spatial environment, while place cells with precise place fields generally preserve spatial information when their fields reappear.

## Significance Statement

Spatial representations in the hippocampus support spatial memory and are comprised of place cells and their corresponding place fields (PFs). Recent work has revealed that spatial representations are not as stable as once thought. Instead, they undergo large changes across days, a phenomenon called representational drift. We present results showing an association between the spatial precision of place fields on 1 d and their drift across subsequent days. We find this association holds true when spatial representations drift in response to changes in reward expectation. We discuss a synaptic-level conceptual model that links place field precision with drift. This study advances our understanding of the mechanisms behind representational drift, providing insight into how the hippocampus updates spatial memories for continual learning.

## Introduction

The hippocampus is known to play a role in encoding, consolidating, updating and retrieving episodic memories (Andersen et al., 2006). Within the hippocampus, there are subsets of cells known as place cells, which exhibit spatial activity patterns corresponding to the animal’s location within a specific environment (J. O’Keefe and Dostrovsky, 1971). These locations are referred to as place fields (PFs), and as a population they provide a spatial representation of a given environment. The faithful reinstatement of hippocampal representations is thought to support memory retrieval (J.N. O’Keefe, 1978; Gelbard-Sagiv et al., 2008; Liu et al., 2012; Josselyn et al., 2015; Frankland et al., 2019). However, recent findings show that spatial representations change with time and experience even when animals are navigating the same environment (Hainmueller and Bartos, 2018; J.S. Lee et al., 2020; Mau et al., 2020; Dong et al., 2021; Driscoll et al., 2022; Keinath et al., 2022). This phenomenon is known as representational drift and can occur during navigation of an environment from lap-to-lap, as demonstrated by many PFs shifting backwards (Mehta et al., 2000; Dong et al., 2021; Roth et al., 2012), and across repeated exposures (epochs of experience) to the same environment on different days (Ziv et al., 2013; Dong et al., 2021). Representational drift may track time (Mankin et al., 2012, 2015; Rubin et al., 2015) or amount of experience (Geva et al., 2023; Khatib et al., 2023). Similar changes to the spatial code are observed when animal’s undergo an internal state change during navigation, as demonstrated when attention or reward expectation is altered in an unchanging spatial environment (Krishnan et al., 2022; Pettit et al., 2022). At the single cell level, the fate of preexisting place fields falls into one of three categories. First, place cells can remap their PFs to new locations. Second, PFs can vanish. Third, place fields can remain stable. However, what determines the fate of PFs across time or internal state changes remains unclear.

To investigate this, we reanalyzed previously published data, where two-photon Ca ${}^{2+}$ imaging was used to record the activity of large populations of pyramidal neurons in dorsal CA1 in head-fixed mice. Mice were placed on a treadmill and repeatedly traversed a virtual linear environment for water rewards. We defined cells with significant PFs during a block of trials in a single session and measured their lap-to-lap properties such as their spatial precision, firing rate variability, and backward shifting. We then determined PF fate in the same environment in either a subsequent block of trials separated by a day (a distinct temporal epoch) or a subsequent block of trials during the same session but with reward removed to change reward expectation (a distinct internal state epoch). Our findings reveal that Remapped PFs across internal state or temporal epochs tended to have lower spatial precision during the initial epoch, whereas Stable and Vanished PFs were associated with high spatial precision. This suggests that place cells with imprecise place fields generally possess greater spatial flexibility, providing a means for the hippocampus to respond to distinct epochs of experience and update spatial representations with new spatial information. Place cells with precise place fields, when they reappear, generally retain the same spatial information about the environment across epochs.

## Materials and Methods

### Subjects

All experimental and surgical procedures were in accordance with the Animal Care and Use Committee guidelines of The University of Chicago. For this study, 10- to 12-week-old male C57BL/6J wild-type (WT) mice (23–33 g) were individually housed in a reverse 12/12 h light/dark cycle with an ambient temperature of ∼20°C and ∼50% humidity. Male mice were used over female mice because of the size and weight of the headplates (9.1 × 31.7 mm, ∼2 g), which were difficult to firmly attach to smaller female skulls. All training and experiments were conducted during the animal’s dark cycle.

### Mouse surgery and virus injection

Mice were anesthetized (∼1–2% isoflurane) and injected with 0.5 ml of saline (intraperitoneal injection) and ∼0.45 ml of meloxicam (1–2 mg/kg, s.c.). For CA1 population imaging, a small (∼0.5–1.0 mm) craniotomy was made over the hippocampus CA1 (1.7 mm lateral, −2.3 mm caudal of bregma). A genetically encoded calcium indicator, AAV1-CamKII-GCaMP6f (Addgene, #100834) was injected into CA1 (∼75 nl) at a depth of 1.25 mm below the surface of the dura using a beveled glass micropipette. Afterwards, the site was covered up using dental cement (Metabond, Parkell Corporation) and a metal head-plate (9.1 × 31.7 mm, Atlas Tool and Die Works) was also attached to the skull with the cement. Mice were separated into individual cages and water restriction began the following day (0.8–1.0 ml/d). Around 7 d later, mice underwent another surgery to implant a hippocampal window as previously described (Dombeck et al., 2010). Following implantation, the head plate was reattached with the addition of a head ring cemented on top of the head plate which was used to house the microscope objective and block out ambient light. Postsurgery, mice were given 2–3 ml of water per day for 3 d to enhance recovery before returning to the reduced water schedule (0.8–1.0 ml/d).

### Behavior and calcium imaging

We analyzed previously published data.

For the experiment across days (Dong et al., 2021), mice (*n* = 5 in total) were trained to run on a treadmill along a 3-m virtual reality (VR) linear track with 4 μm of water reward delivered at the end of the track [the familiar environment (F)]. Mice (*n* = 3, #1, #2, #3) were then imaged over 2 d in the same environment, without exposure to any novel environment. Calcium activity in CA1 pyramidal neurons (*n* = 1282 neurons) were extracted using customized MATLAB script (Sheffield et al., 2017), with parameters and procedures detailed in (Dong et al., 2021).

For the experiment involving the novel environment (N) switch, mice were trained to run on a treadmill in F, and then on imaging days, the mice (*n* = 3, #3, #4, #5) were introduced to N with different 3D visual cues but the same reward location and track length as F. Note that this novel environment N is N2 in Dong et al. (2021). Also note that mouse #3 was also imaged for the experiment in the familiar environment. Again, calcium activity of CA1 pyramidal neurons (*n* = 1704 neurons) were extracted with the same approach. Table 1 lists the experimental conditions each animal went through and how they were included in (Dong et al., 2021).

**Table 1:** Subjects

Subject #	Imaging sessions	Familiar, 2 d (before any exposure to novelty)	Novelty, 2 d (N2 in Dong et al., 2021)	Included in Dong et al. (2021)?
1	Training: F (imaged) Day 1: F (imaged)	✓	×	Not included. Only imaged in F
2	Training: F (imaged) Day 1: F, N1 (imaged)	✓	×	Included for analysis of F vs N
3	Training: F (imaged) Day 1: F, N1 (imaged) Day 2: F, N2 (imaged) Day 3: N2 (imaged)	✓	✓	Included for analysis of F vs N and also re-exposure to N2
4	Training: F (NOT imaged) Day 1: F, N1 (imaged) Day 2: F, N2 (imaged) Day 3: N2 (imaged)	×	✓	Included for analysis of F vs N and also re-exposure to N2
5	Training: F (NOT imaged) Day 1: F, N1 (imaged) Day 2: F, N2 (imaged) Day 3: N2 (imaged)	×	✓	Included for analysis of F vs N and also re-exposure to N2

**Figure 1.**
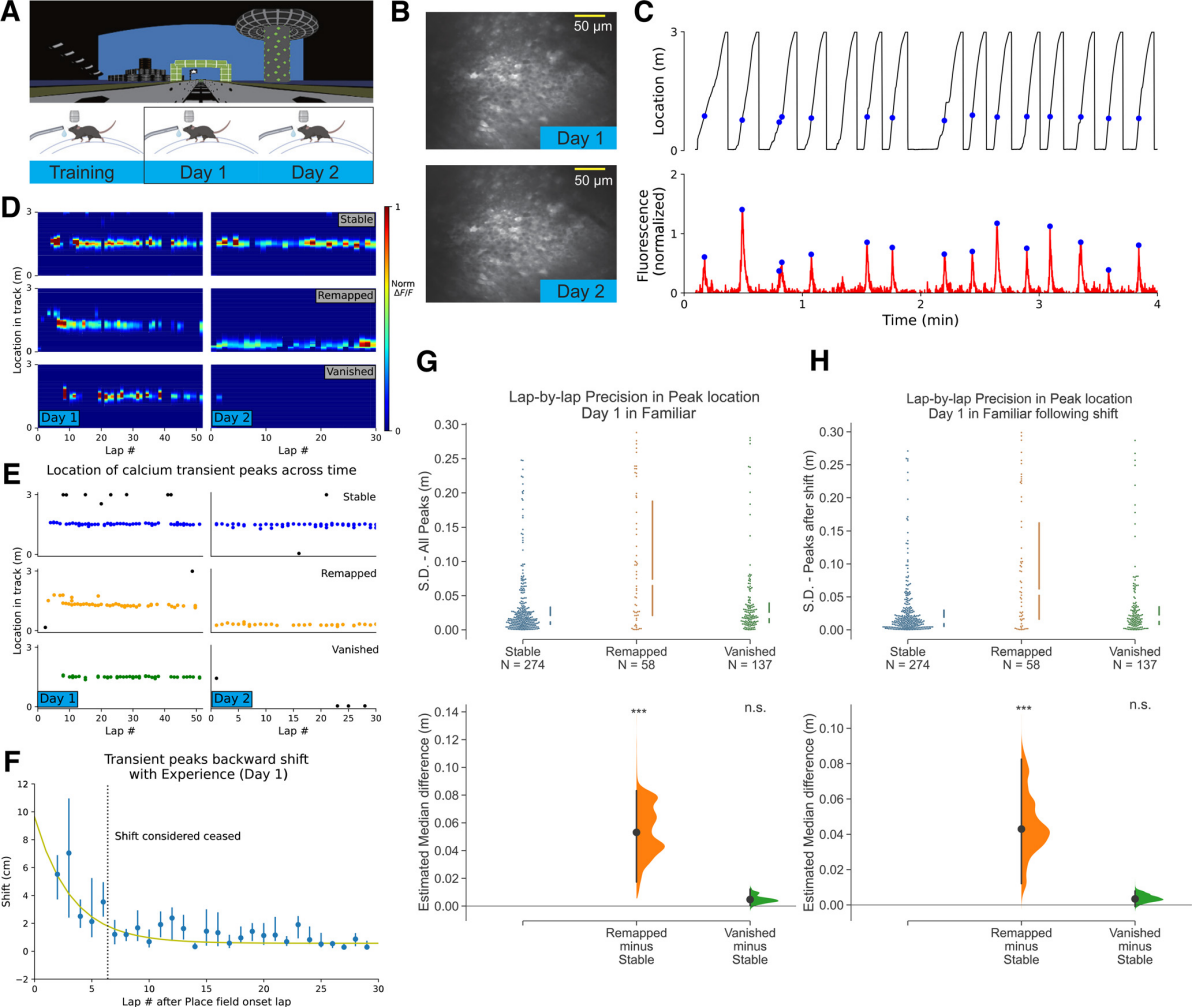
Place fields that remap across 2 d in a familiar environment have lower spatial precision on day 1. ***A***, Experimental design. Top, The familiar virtual reality (VR) environment. Bottom, Animals trained and recorded in the same VR environment. ***B***, Example field of view showing imaging of the same cells on days 1 and 2. ***C***, Top, Behavior of a single animal showing track location. Blue dots indicate Ca^2+^ fluorescence peaks from an example cell relative to the animal’s track location. Bottom, Fluorescence trace of the example cell across time. Blue dots indicating the peak of the Fluorescence change. ***D***, Examples of Stable, Remapped, and Vanished place fields across days. Bins near ends of track are excluded. ***E***, Same as ***D***, but place fields are identified by transient peaks. Colored (blue, orange, and green) dots indicate in-field fluorescence peaks. Black dots are out of field peaks. ***F***, Average population backward shift of PF peaks on day 1. PFs are aligned to their onset lap. Line indicates fitted exponential curve: $F(x)=Ae^{-x/T}$, with A=9.1±4.3 cm, T=3.2±1.1 laps. ***G***, Comparison of spatial precision of PFs (469 PFs in day 1) from the three categories by measuring the SD of the lap-by-lap peak locations of Stable (274/469; 58.4%), Remapped (58/469; 12.4%), and Vanished (137/469; 29.2%) PFs. Medium with inter-quartile range are shown by the broken vertical line next to the each dataset. Bottom, Bootstrapped median difference between the three groups. 5000 resamples, ***p = 4.135E-07 for Remapped versus Stable, *p* = 0.140 for Vanished versus Stable. ***H***, Same as ***G*** but includes only peaks after backward shifting. 5000 resamples, ***p = 2.641E-13 for Remapped versus Stable, *p* = 0.248 for Vanished versus Stable. n.s., not significant.

For multiday imaging datasets, we take an average image at the end of the imaging session on day 1 and use it as a template to find the exact same field of view (FOV) the following day in real time (i.e., before we start collecting data on that day). We match the FOVs within 1 μm of *z*-plane alignment (that is the limit of our microscope controller). We further check alignment by concatenating the FOVs across days and motion correcting them together as one single time-series movie. This corrects for any differences in *X* and *Y* position and allows us to closely inspect any z-differences at the frame transition from day 1 to day 2. If movies from different days are rotated relative to one another, Fiji (ImageJ) is used to correct any rotational displacement between the two movies. We do not use any FOVs that have any noticeable differences in *z*-planes at the transition. All the data shown in this paper are matched in *X*, *Y*, and *Z*. Example of FOVs across days can be found in Figure 1*B*. ROIs are extracted after concatenation. To rule out that the results are not because of imaging artifacts, Extended Data Figure 1-4 shows that there is no correlation between anatomic location of place cells in the FOV and their PF fate. If any imaging artifacts were affecting our PF measurements, the three categories would be equally affected.

10.1523/ENEURO.0261-23.2023.f1-1 Extended Data Figure 1-1 Change in PF locations between blocks of trials. ***A***, Change in PF locations across days in the familiar environment. Median of each category shown in figure. ***B***, Change in PF locations across days in the novel environment. Median of each category shown in figure. ***C***, Change in PF locations between R and UR. Median of each category shown in figure. ***D***, Change in PF locations between R and RR. Median of each category shown in figure. Download Figure 1-1, EPS file.

**Figure 2.**
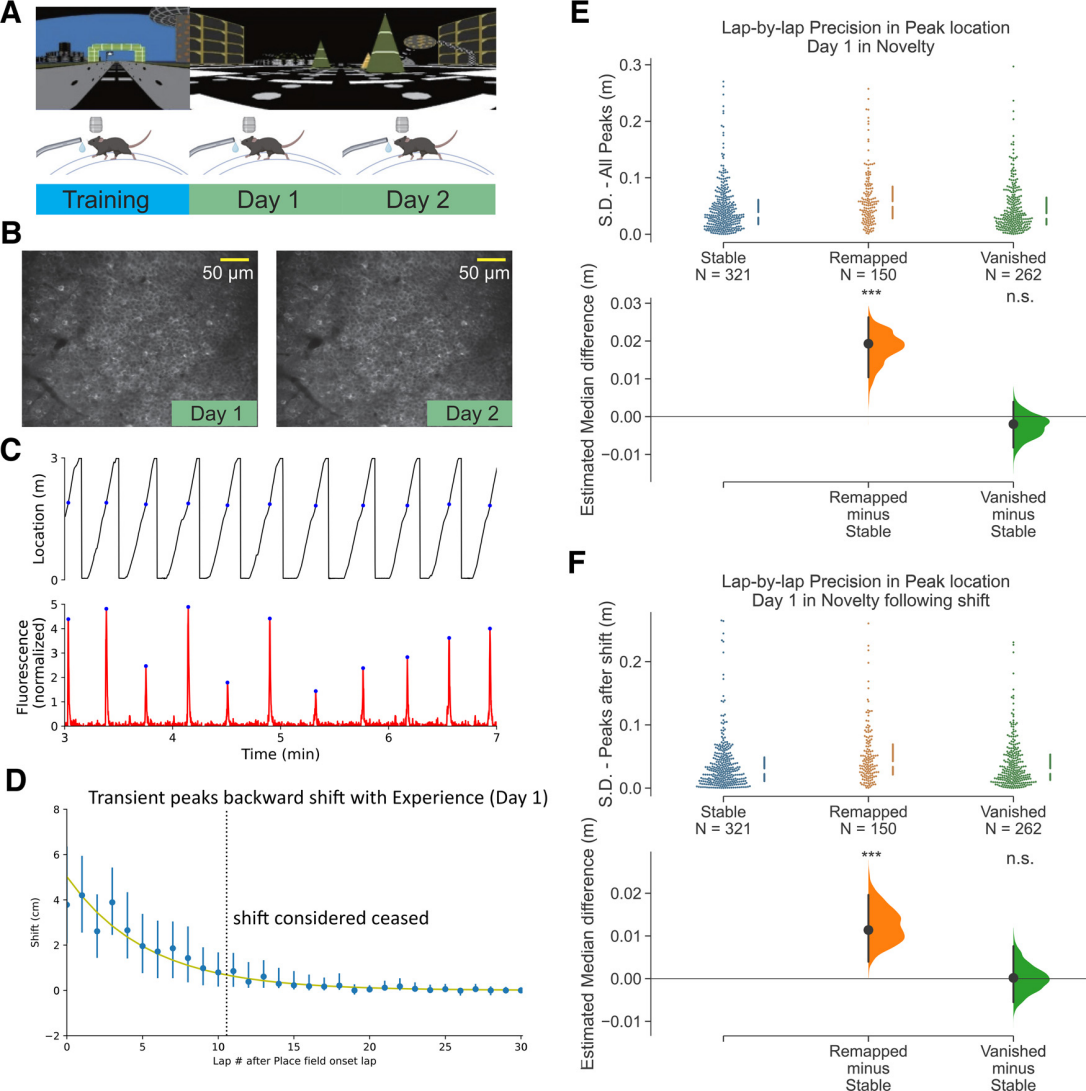
Place fields that remap across 2 d in a novel environment have lower spatial precision on day 1. ***A***, Experimental design. Top, The familiar environment (F) and the novel environment (N). Bottom, The animals were trained in a familiar environment, then switched to a novel environment and imaged for 2 d. ***B***, Example field of view for days 1 and 2 showing the same imaged cells. ***C***, Top, Behavior of a single animal showing track location. Blue dots indicate the animal’s location when the example cell’s calcium transient is at its peak. Bottom, Time-series fluorescent trace for an example cell. Blue dots indicating the transient peaks. ***D***, Average population backward shifting of PF peaks within the session on day 1. PFs are aligned to their onset lap. Line indicates fitted exponential curve: $F(x)=Ae^{-x/T}$, with A=5.0±1.3 cm, T=5.3±1.0 laps. ***E***, Comparison of spatial precision of PFs (733 PFs on day 1) from the three categories by measuring the SD of the lap-by-lap peak locations of Stable (321/733, 43.8%), Remapped (150/733, 20.5%), and Vanished (262/733, 35.7%) PFs. Medium with inter-quartile range are shown by the broken vertical line next to the each dataset. Bottom, Bootstrapped median difference between the three groups. 5000 resamples, ***p = 1.833E-05 for Remapped versus Stable, *p* = 0.383 for Vanished versus Stable. ***F***, Same as ***E*** but includes only peaks after the backward shifting. 5000 resamples, ***p = 0.0006 for Remapped versus Stable, *p* = 0.959 for Vanished versus Stable. n.s., not significant.

10.1523/ENEURO.0261-23.2023.f1-2 Extended Data Figure 1-2 Following the initial epoch, Remapped and Stable PFs have similar spatial precision during subsequent epochs. ***A***, Comparison of spatial precision of PFs on day 2 in the familiar environment. Right, Bootstrapped median difference between Remapped versus Stable PFs from day 1. 5000 resamples, *p* = 0.421. ***B***, Comparison of spatial precision of PFs on day 2 in the novel environment. Right, Bootstrapped median difference between Remapped versus Stable PFs from day 1. 5000 resamples, *p* = 0.811. ***C***, Comparison of spatial precision of PFs in UR condition. Right, Bootstrapped median difference between Remapped versus Stable PFs from R. 5000 resamples, *p* = 0.869. ***D***, Comparison of spatial precision of PFs in RR condition. Right, Bootstrapped median difference between Remapped versus Stable PFs from R. 5000 resamples, *p* = 0.848. Download Figure 1-2, EPS file.

**Figure 3.**
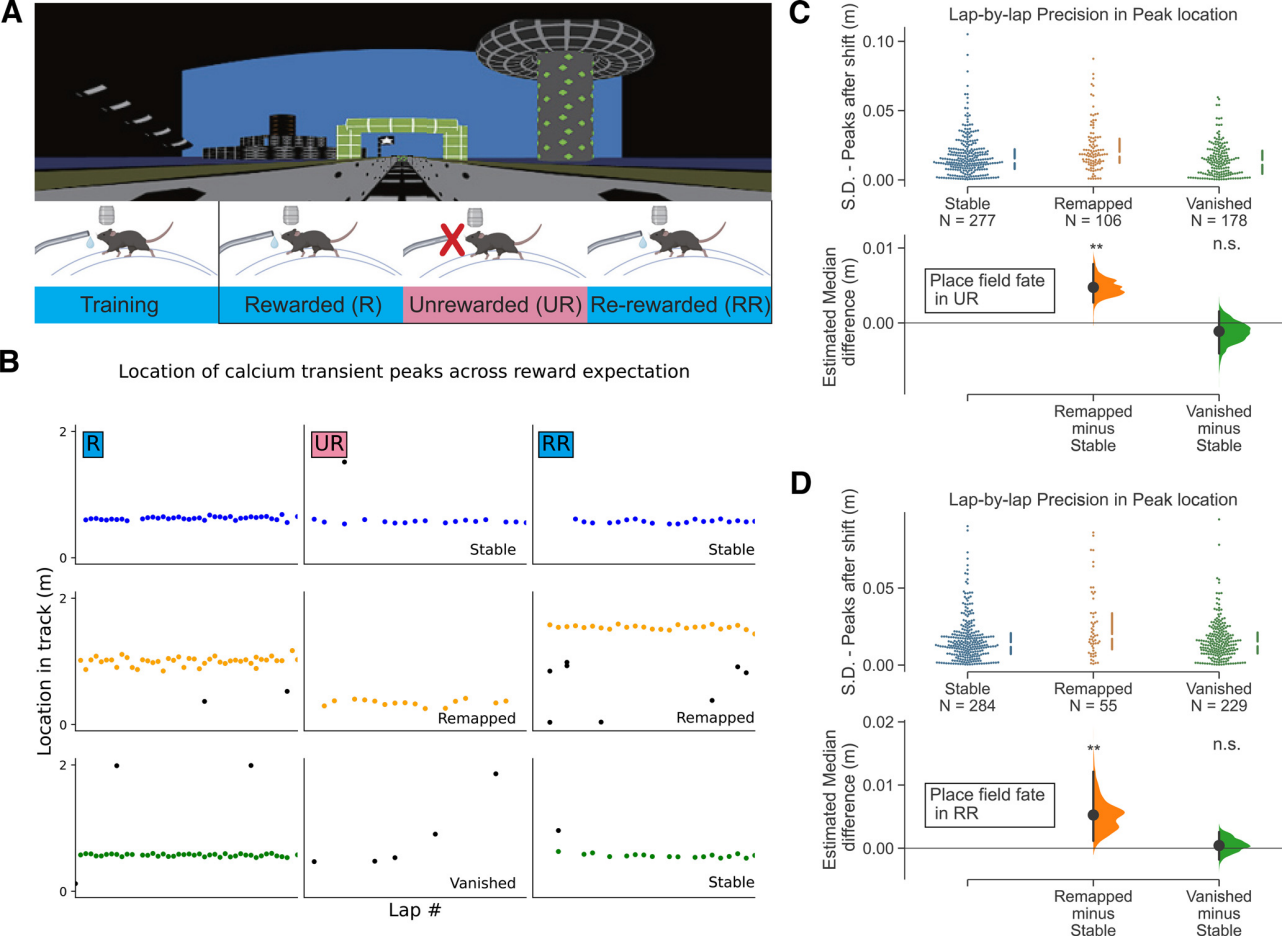
Place fields that remap in response to changed reward expectation tend to have lower spatial precision before the change. ***A***, Experimental design. Top, The familiar VR environment. Bottom, The Animals were trained and recorded in the same VR environment during changes in reward. ***B***, Example of Stable (top), Remapped (middle), and Vanished (bottom) place fields across changes in reward expectation. Colored (blue, orange, and green) dots indicate in-field transient peaks. The place field can undergo remapping in UR and then again in RR. ***C***, Comparison of spatial precision of PFs (561 PFs in R; see Materials and Methods, Defining PFs) from the three categories by measuring the SD of the lap-by-lap peak locations of Stable (277/561, 49.4%), Remapped (106/561, 18.9%), and Vanished (178/561, 31.7%) PFs between R and UR. Medium with inter-quartile range are shown by the broken vertical line next to the each dataset. Bottom, Bootstrapped median difference between the three groups. 5000 resamples, **p = 0.0016 for Remapped versus Stable, *p* = 0.480 for Vanished versus Stable. ***D***, Same as ***C***, but comparison between R and RR. (568 PFs in R, see Materials and Methods, Defining PFs; Stable PFs: 284/568, 50.0%; Remapped PFs: 55/568, 9.7%; Vanished PFs: 229/568, 40.3%) 5000 resamples, **p = 0.0022 for Remapped versus Stable, *p* = 0.683 for Vanished versus Stable. R, Rewarded; UR, Unrewarded; RR, Re-rewarded; n.s., not significant.

10.1523/ENEURO.0261-23.2023.f1-3 Extended Data Figure 1-3 Comparison of median spatial precision of place fields for individual animals. ***A***, Median spatial precision (as measured by the SD of PF peaks) in each category for individual animals in the familiar environment. Significant differences between PF categories are shown using one-way ANOVA (*F* = 8.814, *p* = 0.0164), Tukey’s *post hoc* test shows a significant difference for Stable versus Remapped (*p* = 0.0218), and for Remapped versus Vanished (*p* = 0.0300). ***B***, Same as ***A*** but for novel environment. Significant differences between PF categories are shown using one-way ANOVA (*F* = 8.43, *p* = 0.01810), Tukey’s *post hoc* test shows a significant difference for Stable versus Remapped (*p* = 0.0394), and for Remapped versus Vanished (*p* = 0.0212). ***C***, Same as ***A*** but for the Rewarded (R)-Unrewarded (UR) experiment. PFs are categorized by their fate in UR but their spatial precision is measured in R. Significant differences between PF categories is shown using one-way ANOVA (*F* = 14.24, *p* = 0.000679), Tukey’s *post hoc* test shows a significant difference for Stable versus Remapped (*p* = 4.15E-03), and for Remapped versus Vanished (*p* = 7.97E-04). ***D***, Same as ***A*** but for the Rewarded (R)-re-rewarded (RR) experiment. PFs are categorized by their fate in RR following an epoch of UR, but their spatial precision is measured in R. Significant differences between PF categories is shown using one-way ANOVA (*F* = 21.05, *p* = 0.000119), Tukey’s *post hoc* test shows a significant difference for Stable versus Remapped (*p* = 2.569E-04), and for Remapped versus Vanished (*p* = 3.71E-04). Download Figure 1-3, EPS file.

10.1523/ENEURO.0261-23.2023.f1-4 Extended Data Figure 1-4 Anatomical location of place cells color-coded based on their PF fate in three example animals. ***A***, Anatomical location in x and y of place cells [relative to center of field of view (FOV)] in one example animal (#3, imaged across 2 d in familiar environment). Origin represents center of FOV. ***B***, Comparison of anatomical location of place cells from center of FOV grouped by PF fate category (Stable, *N* = 108; Remapped, *N* = 32; Vanished, *N* = 87). One-way ANOVA shows no statistically significant difference between categories (*F* = 1.160, *p* = 0.315). ***C***, Same as ***A*** but for a different animal (#4, imaged across 2 d in novel environment). ***D***, Same as ***B*** but for animal #4 (Stable, *N* = 176; Remapped, *N* = 82; Vanished, *N* = 170). One-way ANOVA shows no statistically significant difference between categories (*F* = 0.157, *p* = 0.855). ***E***, Same as ***A*** but for a different animal [imaged across blocks of trials with changing reward (R-UR-RR)]. PFs from R are categorized by their fate in UR. ***F***, Same as ***B*** but for the animal in ***E*** (Stable, *N* = 68; Remapped, *N* = 32; Vanished, *N* = 46). One-way ANOVA shows no statistically significant difference between categories (*F* = 1.896, *p* = 0.154). Download Figure 1-4, EPS file.

10.1523/ENEURO.0261-23.2023.f1-5 Extended Data Figure 1-5 Other place field metrics are not associated with place field fate across days in a familiar environment. ***A***, Comparison of backward shifting dynamics between the different PF fate categories. Fitting parameters for the exponential $F(x)=Ae^{-x/T}$: for shift of all PFs: A=9.1±4.3, T=3.2±1.1; for Stable PFs: A=9.4±6.7, T=3.0±1.4; for Remapped PFs: A=7.1±3.9, T=3.6±1.3; for Vanished PFs: A=8.0±5.3, T=3.7±1.5. ***B***, Comparison of median of peak amplitude. *p* = 0.577 for Stable versus Remapped, 5000 resamples, *p* = 0.210 for Stable versus Vanished. ***C***, Comparison of lap-by-lap peak amplitude variation. *p* = 0.313 for Stable versus Remapped, 5000 resamples, *p* = 0.915 for Stable versus Vanished. ***D***, Histograms of PF onset laps, end laps, and duration (in laps) for Stable, Remapped, and Vanished PFs. Cumulative fraction plots (right). Wilcoxon rank-sum test, for Stable versus Remapped: start time: *p* = 0.160, end time: *p* = 0.815, time length: *p* = 0.918; for Stable versus Vanished: start time: *p* = 0.884, end time: *p* = 0.907, time length: *p* = 0.941; for Remapped versus Vanished: start time: *p* = 0.173, end time: *p* = 0.953, time length: *p* = 0.965. Download Figure 1-5, EPS file.

10.1523/ENEURO.0261-23.2023.tab1-1 Extended Data Table 1-1 **Statistics** Download Table 1-1, XLSX file.

For the experiment with change in reward contingencies (Krishnan et al., 2022), mice were trained on a 2-m VR linear track for water reward. Well-trained mice showed preemptive licking before the reward location. On experimental day, the mice (*n* = 5) ran in the environment with reward (R), then the reward was unexpectedly removed (UR). The reward was then reintroduced (RR). Each condition (R, UR, and RR) lasted 8–10 min. Population activity of CA1 pyramidal neurons (*n* = 1288) were measured with Ca ${}^{2+}$ imaging, across the conditions. Calcium transients were extracted using suite2p (Pachitariu et al., 2017) as in (Krishnan et al., 2022).

### Defining PFs

After extracting significant calcium transients, we correlated the transients to the animals’ behavior. We obtained the significant peak of each transient by finding the local maximum of a transient that exceeds the mean ΔF/F by three interquartile range of ΔF/F in a time window of 20 frames, to avoid including peaks from noise. The corresponding animal location on the track were then obtained. Fluorescence peaks were treated as events in a 2D parameter space (time and location). PFs were then defined by event clusters in the 2D parameter space using the clustering algorithm DBSCAN (Ester et al., 1996), a density based clustering algorithm. A cluster then needed to include events from at least 10 different laps to be considered a PF.

The vast majority of cells had either a single cluster or no cluster in any given epoch. In the limited number of cells that had multiple clusters in either epoch, each cluster was treated as independent PF. For the very few cells with multiple clusters in epoch 1 and epoch 2, the clusters across the epochs were “paired,” such that clusters in epoch 1 were paired with the closet ones in epoch 2 and analyzed as such. In cases with two clusters in epoch 1 and one cluster in epoch 2, we considered the clusters to have merged across epochs If both clusters in the first epoch were within 40 cm of the epoch 2 cluster (if one or both were not, we did not consider them merged and treated each independently). The two clusters in epoch 1 were therefore counted as one Stable PF, and we combined their spatial precision values (see PF properties on how we did this), to avoid overcounting the Stable PFs. This caused the difference in total number of PFs in R for Figure 3*C*,*D*, because of cells with multiple clusters in R having different fates in UR and RR.

### PF properties

Once clusters were identified as PFs, the time, location, and transient peak ΔF/F for each event within the cluster were quantified. To determine the onset and offset of the PF, we defined the PF onset lap as the lap number of the first event in the cluster, and the PF offset lap as the lap number of the last event in the cluster. The duration of the PF was then calculated as the difference between the onset and offset lap.

The spatial location of the PF was defined as the median of the locations of the events within the cluster. The spatial precision of the PF was quantified using the SD of the location in the cluster. Therefore, a precise PF means a lower lap-to-lap variation in firing location, and hence a lower SD. To ensure that this measurement was not influenced by the edges of the track, PFs located within 10 cm of the track ends were excluded from the analysis.

In the rare cases where two PFs merged into a single PF across epochs (see above, Defining PFs), we combined their precision measure into a single value and only counted it as a single Stable PF. To obtain a single precision value from the two PFs, we obtained the location of each event from each cluster and then subtracted the mean location of that cluster from each event $\left(x_{i}-\bar{x}\right)$. Then, the two mean-removed sets of locations were merged and the spatial precision was measured by SD of the merged set. This was performed to determine the combined spatial precision of the two PFs, that we considered a single Stable PF for this analysis.

The PF firing rate dynamics were investigated by using the ΔF/F peak amplitude as a proxy for the maximum firing rate. The lap-by-lap firing rate dynamics were measured by calculating the deviation in the peak amplitude of events within the cluster.

### PF categorization

To assess the spatial stability of PFs, we categorized them based on their change in spatial location across days/conditions:

1. Stable: the PF is present on both days/conditions and any change in PF position is: Δ<40 cm.
2. Remapped: the PF is present on both days/conditions but changes PF position: Δ>40 cm.
3. Vanished: The PF is only present on the first day/condition.

The specific choice of 40 cm as the threshold was made to ensure that it was larger than the typical fluctuation observed in peak locations within clusters in the dataset (a measure of PF width). This means that a change in location beyond 40 cm would typically mean a nonoverlapping PF.

### PF backward shifting

PFs were aligned by their onset lap, which was defined as the lap number of the first peak in that cluster. Then the spatial positions of the PFs on each lap were obtained with a sliding window of five laps. The five-lap sliding average position of individual PFs were then compared with the median location calculated from laps beyond the 15th lap from the onset lap for that PF. For each lap from the PF onset lap, the average shift over the population of PFs was calculated and plotted. An exponential fit (least square fit, scipy.optimize.curve_fit) was then applied to the trend. The error of the fitting parameters was obtained by the square-root of the diagonal elements of the covariance matrix (returned by curve_fit). The same trend was observed without the smoothing.

PFs were considered to have ceased systematically backward shifting after 2*T* laps from their onset, as the decay in shift is reduced to $e^{-2}=13.5\%$ at 2*T*. Peaks after 2*T* laps were included in the comparisons that restrict to PF activity following backward shifting. Similar trends were observed using different choices than 2*T* for the cutoff.

### Statistics

For the plots regarding PF backward shifting, the error bars represent mean ± SEM.

To generate the plots comparing the spatial precision, we used the package DABEST (data analysis with bootstrap-coupled estimation; Ho et al., 2019). As explained in PF properties, this gives a measure of the precision of PFs in each category. To compare the difference in the population, the median difference between the distributions and its confidence level were obtained with bootstrapping (5000 resamples); *p*-values of the nonparametric two-sided approximate permutation *t* test were reported. See Extended Data Table 1-1 for the specific statistical tests used in each figure.

Schematic figures (Figs. 1*A*, 2*A*, 3*A*) were created with BioRender.

10.1523/ENEURO.0261-23.2023.f2-1 Extended Data Figure 2-1 Other place field metrics are not associated with place field fate across days in a novel environment. ***A***, Comparison of backward shifting dynamics between the different categories of PF fate. Fitting parameters for the exponential $F(x)=Ae^{-x/T}$: for shift of all PFs: A=5.0±1.3, T=5.3±1.0; for Stable PFs: A=4.6±1.0, T=5.0±0.9; for Remapped PFs: A=6.2±1.8, T=4.5±0.9; for Vanished PFs: A=5.1±1.3, T=5.4±1.0. ***B***, Comparison of median of peak amplitude. *p* = 0.259 for Stable versus Remapped, 5000 resamples, *p* = 0.094 for Stable versus Vanished. ***C***, Comparison of lap-by-lap peak amplitude variation. *p* = 0.223 for Stable versus Remapped, 5000 resamples, *p* = 0.847 for Stable versus Vanished. ***D***, Histograms of PF onset laps, end laps, and duration (in laps) for Stable, Remapped, and Vanished PFs. Cumulative fraction plots (right). Wilcoxon rank-sum test, for Stable versus Remapped: start time: *p* = 0.474, end time: *p* = 0.704, time length: *p* = 0.883; for Stable versus Vanished: start time: *p* = 0.589, end time: *p* = 0.616, time length: *p* = 0.930; for Remapped versus Vanished: start time: *p* = 0.759, end time: *p* = 0.953, time length: *p* = 0.998. Download Figure 2-1, EPS file.

10.1523/ENEURO.0261-23.2023.f3-1 Extended Data Figure 3-1 Other place field metrics in R are not associated with place field fate in UR. ***A***, Comparison of backward shifting dynamics between the different categories of PF fate. Fitting parameters for the exponential $F(x)=Ae^{-x/T}$: for shift of all PFs: A=7.0±1.2, T=3.4±0.7; For Stable PFs: A=7.1±1.3, T=3.2±0.7; for Remapped PFs: A=6.1±1.7, T=3.8±1.1; for Vanished PFs: A=6.4±1.6, T=3.8±1.1. ***B***, Comparison of median of peak amplitude. *p* = 0.437 for Stable versus Remapped, 5000 resamples, *p* = 0.2472 for Stable versus Vanished. ***C***, Comparison of lap-by-lap peak amplitude variation. *p* = 0.140 for Stable versus Remapped, 5000 resamples, *p* = 0.996 for Stable versus Vanished. ***D***, Histograms of PF onset laps, end laps, and duration (in laps) for Stable, Remapped, and Vanished PFs. Cumulative fraction plots (right). Wilcoxon rank-sum test, for Stable versus Remapped: start time: *p* = 0.475, end time: *p* = 0.792, time length: *p* = 0.850; for Stable versus Vanished: start time: *p* = 0.759, end time: *p* = 0.977, time length: *p* = 0.804; for Remapped versus Vanished: start time: *p* = 0.511, end time: *p* = 0.770, time length: *p* = 0.965. Download Figure 3-1, EPS file.

10.1523/ENEURO.0261-23.2023.f3-2 Extended Data Figure 3-2 Other place field metrics in R are not associated with place field fate in RR. ***A***, Comparison of backward shifting dynamics between the different categories of PF fate. Fitting parameters for the exponential $F(x)=Ae^{-x/T}$: for shift of all PFs: A=6.6±1.2, T=3.6±0.8; for Stable PFs: A=7.1±0.9, T=3.8±0.7; for Remapped PFs: A=6.8±0.8, T=3.9±0.7; for Vanished PFs: A=6.7±2.0, T=3.5±1.0. ***B***, Comparison of median of peak amplitude. *p* = 0.567 for Stable versus Remapped, 5000 resamples, *p* = 0.466 for Stable versus Vanished. ***C***, Comparison of lap-by-lap peak amplitude variation. *p* = 0.338 for Stable versus Remapped, 5000 resamples, *p* = 0.680 for Stable versus Vanished. ***D***, Histograms of PF onset laps, end laps, and duration (in laps) for Stable, Remapped, and Vanished PFs. Cumulative fraction plots (right). Wilcoxon rank-sum test, for Stable versus Remapped: start time: *p* = 0.737, end time: *p* = 0.884, time length: *p* = 0.965; for Stable versus Vanished: start time: *p* = 0.650, end time: *p* = 0.965, time length: *p* = 0.895; for Remapped versus Vanished: start time: *p* = 0.965, end time: *p* = 0.838, time length: *p* = 0.988. Download Figure 3-2, EPS file.

## Results

### Place fields that remap across 2 d in a familiar environment have lower spatial precision on day 1

To address whether place field characteristics during a single epoch of navigation in an environment were associated with their fate during a second epoch of the environment, head-fixed mice (*n* = 3) were trained to navigate a familiar VR environment (Fig. 1*A*) while the same populations of place cells were imaged in CA1 during two blocks of trials (distinct temporal epochs) separated by a day (Fig. 1*B*). The peak Ca^2+^ fluorescence on each lap traversal was used as a proxy for maximum spatial firing position on each lap. Peaks were then treated as events in 2D parameter space (time and spatial position). Clusters of events with consistent spatial position were identified as PFs (see Materials and Methods). The mean spatial position was then calculated and the same analysis was done the following day. PFs calculated on day 1 were then defined as either Stable, Remapped, or Vanished based on their mean activity on day 2 (see PF categorization and Extended Data Fig. 1-1). PFs were considered Remapped if the change in PF location across days was >40 cm (for why this threshold was chosen, see Materials and Methods). Note that in this paper we distinguish between PFs that change spatial position (referred as “Remapped”) and PFs that disappear (referred as “Vanished”; see examples in Fig. 1*D*). The median change in spatial position across days in the Stable PF group was 2.6 versus 179.8 cm in the Remapped group (Extended Data Fig. 1-1*A*).

Various properties of individual PFs in each category were then analyzed on day 1. First, lap-wise spatial precision was quantified using the SD of spatial locations of the fluorescent peaks. We compared the spatial precision of PFs across the three categories (Fig. 1*F*). The PFs that remapped on day 2 exhibited significantly lower spatial precision on day 1, as revealed by a higher variation in lap-to-lap PF position (Median (Mdn) SD = 6.9 cm, Inter-quartile range (IQR) = 2.1–18.8 cm) than Stable PFs (Mdn SD = 1.6 cm, IQR = 0.8–3.3 cm; *p* = 4.135E-07). Vanished PFs, however, exhibited similar spatial precision to Stable PFs (Mdn SD = 2.2 cm, IQR = 1.1–3.9 cm; *p* = 0.14).

Studies have reported a type of drift on a lap-by-lap basis that occurs during navigation, also known as PF backward shifting (Mehta et al., 2000; I. Lee and Knierim, 2007; Roth et al., 2012; Dong et al., 2021; Geva et al., 2023; Khatib et al., 2023). Backward shifting could reduce the lap-wise precision of PFs as we measured it here. To determine whether backward shifting contributed to our measure of precision and its association with PF fate, we measured the extent of backward shifting on day 1 (Fig. 1*E*). Further, because not all PFs emerge immediately when mice start navigating a familiar environment on any particular day, we first defined the PF onset lap for each PF (Sheffield et al., 2017; Dong et al., 2021). Aligning PFs to their onset lap, we found that backward shifting ceased after a finite number of laps, and the decay of shifting could be well fitted to an exponential (Fig. 1*E*). We estimated the time constant *T* of the decay. We considered PFs to have ceased backward shifting after 2*T* laps from their onset, as this is the point at which 90% of the shifting had decayed (see PF backward shifting). Then, the same comparison was performed as Figure 1*F*, but restricted to PF activity following the backward shifting. We found that the association between PF precision and PF fate across days was maintained even when backward shifting on day 1 was excluded from the analysis Figure 1*G* (Stable PFs: Mdn SD = 1.4 cm, IQR = 0.5–3.0 cm; Remapped PFs: Mdn SD = 5.7 cm, IQR = 1.6–16.2 cm; Vanished PFs: Mdn SD = 1.7 cm, IQR = 0.8–3.5 cm). This was also true for individual animals, shown in Extended Data Figure 1-3*A*.

It is possible that cells closer to the center of the imaging field of view (FOV) are of higher image quality, i.e., are less sensitive to imaging artifacts which could introduce noise. Cells further from the center of the FOV could therefore produce noisier signals that could make their PFs appear less precise and also make them more difficult to detect across days, making them appear less stable. To test whether this potential artifact is driving the association between PF precision and PF fate, we correlated the anatomic location of place cells in the FOV with their PF fate, which is shown in Extended Data Figure 1-4 for three example animals. We found no correlation between the location of place cells in the FOV and the fate of their PFs across days, ruling out any imaging artifacts driving the association between PF precision and fate.

We next checked whether the Remapped and Stable PFs continued to have differences in spatial precision on day 2 (Extended Data Fig. 1-2*A*). We found no such difference, showing that on day 2, the PFs that had remapped and the PFs that had stabilized have the same median precision. This suggests that PFs can switch from one category to the other. In other words, an imprecise PF that remaps across a day can then become precise and therefore stable across a subsequent day, and vice versa. While the spatial precision on day 1 is relevant to PF fate across a day, we next tested whether other PF properties on day 1 were associated with PF fate. We first asked whether the extent of backward shifting of PFs was associated with their fate. In Extended Data Figure 1-5*A*, we show the lap-by-lap shifting of all the PFs, and separately, the PFs from each category fitted to an exponential. Remapped, Stable, and Vanished PFs showed similar backward shifting dynamics. Comparing both the amplitudes and the time constants for the exponential fits along with their uncertainty values for all the categories demonstrated no differences between the groups (Extended Data Fig. 1-5*A*).

Next, we asked whether PF firing rates were associated with PF fate. Using peak amplitudes of calcium transients as a proxy for max firing rate on each PF traversal, we quantified the median and deviation in amplitudes from lap-to-lap. Comparing this measure between the three categories of PF fate revealed no significant difference (Extended Data Fig. 1-5*B*,*C*).

Not only do PFs emerge on different laps in a familiar environment, place cells can stop firing in their PF before the session ends. The PF onset lap and PF end lap, as well as the total laps in between onset and end (PF duration), can therefore be quantified for each PF. Extended Data Figure 1-5*D* shows the histograms of PF onset laps, end laps and total laps for the three PF fate categories. We found no differences between the PF fate categories.

Together, our investigation into place field properties and PF fate across days in a familiar environment suggests that it is randomly varying lap-by-lap spatial dynamics on day 1 that is related to the across-day fate of PFs, and other PF properties are unrelated.

### Place fields that remap across 2 d in a novel environment have lower spatial precision on day 1

When mice are introduced to a novel environment, global remapping occurs in CA1 in which a new map forms (Colgin et al., 2008; Sheffield et al., 2017; Dong et al., 2021). Once the PFs that comprise the new map emerge, they typically are less precise than in familiar environments (Frank et al., 2004). We therefore tested whether the relationship between lap-wise precision and across-day PF fate that we observed in a familiar environment also occurred in a novel environment during familiarization. We therefore switched mice (*n* = 3) to a novel VR environment while imaging CA1 (Fig. 2*A*,*B*) and identified PFs (Fig. 2*C*). We first wanted to determine how the newly-formed PF map backward shifted from lap-to-lap on day 1 (Fig. 2*D*). We found backward shifting was prolonged compared with the familiar environment ( T=3.2±1.1 laps for familiar environment and T=5.3±1.0 laps for novel environment) as previously reported (Dong et al., 2021). We then measured PF precision on day 1 and compared between the three categories of PFs based on their fate on day 2. Once again, we observed that Remapped PFs had lower spatial precision than Stable and Vanished PFs as shown by having a higher median SD (Fig. 2*F*; Remapped PFs: Mdn SD = 5.4 cm, IQR = 2.8–8.4 cm; Stable PFs: Mdn SD = 3.4 cm, IQR = 1.7–6.1 cm; Vanished PFs: Mdn SD = 3.2 cm, IQR = 1.7–6.5 cm), even when backward shifting was excluded from the precision analysis (Fig. 2*G*; Remapped PFs: Mdn SD = 3.8 cm, IQR = 2.1–6.9 cm; Stable PFs: Mdn SD = 2.7 cm, IQR = 1.1–4.9 cm; Vanished PFs: Mdn SD = 2.7 cm, IQR = 1.3–5.3 cm). Also, just as in a familiar environment, day 2 lap-by-lap precision showed no difference between Remapped and Stable PFs (Extended Data Fig. 1-2*B*), and no other PF properties measured on day 1 were related to PF fate on day 2 (Extended Data Fig. 2-1).

### Place fields that remap in response to changed reward expectation tend to have lower spatial precision before the change

A recent study showed that some PFs remap when the internal state of reward expectation changes in an unchanging spatial environment (Krishnan et al., 2022). We therefore asked whether lap-by-lap spatial precision of PFs was associated with remapping under these conditions of altered internal state. To do this, mice were trained and then imaged in the same familiar rewarded environment (Fig. 3*A*). Trained mice (*n* = 5) were first water rewarded for a block of trials and then reward was removed for a subsequent block of trials [unrewarded condition (UR)]. After a few laps, mice stopped preemptively licking for reward, demonstrating a loss of reward expectation (Krishnan et al., 2022). Then, reward was reintroduced [rerewarded condition (RR)].

Figure 3*B* shows example place cells; one with a Stable PF across the R-UR-RR conditions (top), one with a Remapped PF across all conditions (middle), and one PF that vanished in UR but reappeared in RR (bottom). We then investigated whether the lap-by-lap spatial precision of PFs in R determined their fate in UR or RR (Fig. 3*C*,*D*). Similar to the fate of PFs across days, we found that the Remapped PFs in UR exhibited lower spatial precision in R (Fig. 3*C*; Remapped PFs: Mdn SD = 1.9 cm, IQR = 1.2–3.0 cm; Stable PFs: Mdn SD = 1.4 cm, IQR = 0.8–2.2 cm; Vanished PFs: Mdn SD = 1.3 cm, IQR = 0.4–2.1 cm) and RR (Fig. 3*D*; Remapped PFs: Mdn SD = 2.0 cm, IQR = 1.2–3.4 cm; Stable PFs: Mdn SD = 1.3 cm, IQR = 0.8–2.2 cm; Vanished PFs: Mdn SD = 1.2 cm, IQR = 0.5–1.9 cm). Again, other PF properties had no association with remapping or stability (Extended Data Figs. 3-1 and 3-2). This indicates that across the population, Remapped PFs caused by internal state changes tend to have lower precision than Stable/Vanished PFs, behaving similarly to Remapped PFs across days.

## Discussion

We investigated hippocampal CA1 spatial code dynamics occurring across epochs in unchanging spatial environments to determine whether firing characteristics during a single epoch was associated with how cells encode future epochs. Our findings show that PFs that remapped across epochs separated in time by a day, or across epochs distinguished by differences in reward expectation, had a statistically lower lap-by-lap spatial precision during the initial epoch, compared with Stable and Vanished PFs. This held true across epochs in novel environments as mice underwent familiarization (a form of learning). Other lap-by-lap characteristics of PFs, such as firing rate variability, backward shifting dynamics, PF onset, PF offset, and PF duration were not associated with the fate of PFs across epochs. This indicates that the spatial firing precision of PFs during navigation is related to their tendency to remap or stabilize/vanish across distinct epochs of experience.

Drift across time has been observed in different parts of the brain (Driscoll et al., 2017; Deitch et al., 2021; Marks and Goard, 2021; Schoonover et al., 2021). In the hippocampus, an accurate representation of the spatial environment is preserved during drift (Ziv et al., 2013; Keinath et al., 2022), suggesting drift may encode nonspatial factors of the context such as time (Mankin et al., 2012, 2015), and experience (Geva et al., 2023; Khatib et al., 2023). Our data suggest that the dynamics of drift across epochs is related to the cellular activity within an epoch. This also holds true for epochs that are separated by internal state changes. The dynamics of the hippocampal spatial code across epochs may therefore not be random and may instead be predictable. However, the extent of predictability remains to be directly tested.

A possible explanation for the relationship between lap-by-lap dynamics and the tendency to remap is that those PFs with less spatial precision simply receive higher variability in the activation of the CA3 inputs they receive (Davoudi and Foster, 2019; Devalle and Roxin, 2022; Zutshi et al., 2022). Alternatively, evidence suggests that all CA1 pyramidal cells may receive synaptic input regarding all locations in an environment from CA3 (Grienberger et al., 2017). What determines whether a cell fires at a given location may therefore be the strength of synapses activated at particular locations. Indeed, dendritic spikes in CA1 place cells, which is a reflection of strong synaptic input to a dendritic branch, is associated with PF stability across days (Sheffield and Dombeck, 2015). One idea is that these strong synapses may have undergone Hebbian potentiation, and together with the resultant somatic firing may induce homeostatic mechanisms that lower overall cellular excitability (through synaptic or intrinsic excitability renormalization; Miller, 1996) and make other sets of synapses too weak to cause somatic firing in a winner-takes-all manner (Barry and Burgess, 2007; Sheffield and Dombeck, 2015). This process would result in a precise PF as the cell would fire only in response to those specific inputs. The dendritic spikes associated with this strong input may further serve to maintain synaptic strength to stabilize the PF across days (Sheffield and Dombeck, 2015). On the other hand, PFs with more lap-wise fluctuations in spatial firing may reflect differences in the sets of synapses activated from lap-to-lap. Such variations may not engage Hebbian potentiation and thus avoid the homeostatic winner-takes-all process described above. This would both cause the cell to be less precise from lap-to-lap but also allow the cell more flexibility to respond to new sets of synaptic activation that may occur across distinct epochs of experience, allowing for continuous encoding of new information in the hippocampus.

Our results also show that the PFs that vanished across epochs are indistinguishable from the stable ones in terms of spatial precision. This aligns with previous literature (Ziv et al., 2013), that place cells enter and exit an active subset of an underlying stable map. When these cells are active again, their PFs retain their locations (Ziv et al., 2013). The PFs that vanished across epochs may actually be part of this stable map, they are just not participating in the active subset during a particular epoch. This is likely because of the CA3 inputs that could drive them to fire not being activated.

Overall, our study presents how lap-by-lap dynamics of PFs during an epoch of experience relate to spatial code dynamics across epochs in the same environment. This work provides insight into why some cells remap and others remain stable/vanish. It also provides insight into the synaptic mechanisms which may facilitate these cellular dynamics to support episodic memory encoding.

## Synthesis

Reviewing Editor: Jonathan Lee, University of Birmingham Decisions are customarily a result of the Reviewing Editor and the peer reviewers coming together and discussing their recommendations until a consensus is reached. When revisions are invited, a fact-based synthesis statement explaining their decision and outlining what is needed to prepare a revision will be listed below. The following reviewer(s) agreed to reveal their identity: NONE.

The manuscript was reviewed by 2 reviewers and the Reviewing Editor. We agreed that the paper presents a highly interesting re-analysis of previous data. Establishing a relationship between place cell precision and tendency to remap has the potential to explain at least some of the variability in place cell firing.

Given the potential importance and influence of the interpretation presented in the paper, we feel there is an opportunity to present the data in a more convincing manner, which might involve additional analysis and different presentation of the data. The full reviews are appended below, which provide the complete request, and to which a full response is requested. In brief, it would be beneficial to convince the reader that the observed effect is present within each/most animal(s), which would counter the critical challenge that the effect only is observed due to systematic differences between individual animals. Secondly, a more convincing narrative that the remapping (or otherwise) of place cells is genuinely related to the same cell being recorded over time would be helpful.

*Reviewer 1*

First and foremost, I would like to commend the authors for addressing a crucial aspect of hippocampal place cell dynamics. The study is well-structured, well-quantified and the findings provide valuable insights into the adaptability and flexibility of the hippocampal spatial code. The findings related to the early stage spatial precision of place fields and their tendency to remap or stabilize, even when excluding effects of backward shifting dynamics, are intriguing. It might be helpful to perform more population level analysis and simulation to illustrate the potential function of such a dynamic code in memory encoding and retrieval. Further experiment might be worth trying to solve the causal relationship between the place field precision and remapping tendency.

*Reviewer 2*

The authors present reanalysis of data from two recent studies Dong et al., 2021 and Katib et al., 2023, which include place cell recording from dorsal CA1 made using calcium imaging and virtual reality. The authors observe that the precision of a place cell predicts its upcoming fate in a number of different changes to the context. The precision of the place cell refers to the variance of the peak lap-by-lap location; the fate corresponded to a stable, remapped, or vanished place cell in the three changed contexts. This is an intriguing finding, and suggests that there are common latent factors for each cell that are reflected in these measures. The authors do a good job at accounting for the backwards shift in place field firing, and reporting other characteristics of place fields. Moreover, the authors are to be commended for tackling trial-to-trial variation as a topic of interest, since this information often gets lost in averaging. However, despite this, there are a number of concerns I have with the manuscript. The most important of these is confirmation that the effect is real. My other main concern refers to the source of the data, and inconsistencies with the previously reported animal numbers and conditions. If, these points are addressed, I would have much more confidence in the results, and would have no reservations about recommending publication.

Animal numbers and data reuse

I cannot seem to make the animal numbers agree with the previously published manuscript (Dong et al., 2021). Where there were 4 mice reported for the full F; F N1; F N2 sequence. There are three different cell counts given for different conditions, and I can’t work out which animals went into which one.

As far as I can gather from the methods in the current manuscript the following is reported

DAY1 DAY2

Mouse# Training F N1 F N2

1 + * *

2 + * *

3 + * * * *

4 + * *

5 + * *

Please fully report the a) behavioural sequence for all animals/groups b) the imaging data collected (and if collected and not used, why). A table (such as the one above) and which also includes from which previous publication each animal comes from would probably help future readers.

Presentation of novel vs familiar results separately

If my interpretation of the which animals were recorded from in each condition is correct (see above), I understand why these data have been split up. You only have one animal for which you have HPC recording for the same cells across all the experimental epochs. However, the Dong et al., paper suggests you do have this data (at least for the four animals presented there). The authors need to either explain the discrepancy, OR, provide an analysis which considers the fate of each cell for a both a novel and a familiar day1 vs day2. Ie are cells that are unstable in day1F also unstable in day1N1?

Precision and remapping are potentially confounded by ROI quality

It is not unreasonable to think that an ROI with poor signal-to-noise will show a more variable PF response within a session due to, for instance, higher contamination from other cells (either neuropil or other nearby soma); in a subsequent session, this cell might be easier to mistake for another cell and so seem to be remapped. The authors’ observation that peak amplitudes of calcium transients (and other parameters) was unchanged across groups would suggest this is not the case, but I think it still needs checking. I would suggest that the authors look at a metric of ROI isolation quality and see if it is different between the three cases. A quick-and-dirty check for this would be to use the distance from the center of the FOV as a quality metric proxy, since it looks from your imaging that the center has the best imaging. I won’t specify a metric, as they are often included with whatever ROI finding pipeline one uses, so any reasonable one would do.

Tracking cells over sessions

I did not feel like the cell tracking over days was sufficiently explained. I tried to find details in Dong et al., 2021 and did not find any. Following the references, it seems that the method for ROI extraction is originally from Mukamel et al., 2009, which describes an activity based, ICA method. Are you running the analysis separately for each day? If so, how do you detect vanished cells in day 2? how do you in general perform correspondence across days? The central concern here is that if you don’t see a cell, it could be silent, or the FOV could have shifted so you are not detecting it.

Place field responses over conditions

The authors do not show enough place cell data in their figures, only showing fluorescent traces in time with position above for a single cell for 2 of three figures. This type of plot of is not suitable to assess the precision of a place cell, which underlies the whole result. I would like to see conventional heatmap plats which show the fluorescence for each lap, aligned to the track position (eg Dong et al., fig 2c). I think that showing at least one example from each category (stable, remapped and vanished) would strengthen the presentation of the results.

Effect per animal

I would like to see if the effect is true within animal. One concern with this type of result is that it is driven by some individual difference between animals. For instance, one animal just has less precise place cell response, and more remapping overall due to difference in behavior or something about the imaging or the expression. This would drive a measured difference between the cases if all the cells are treated as independent, as they have been in these analyses. The most conservative way to test for this is to perform separate statistical tests for each animal; an alternative would be to use a mixed effects model with the animal ID being a random term; the minimum I would like to see would be at least the effect going in the same direction for each animal, or if that is not the case, reporting it. This is a common issue in systems neuroscience (see Lazic et al., 2010 BMC Neuroscience).

Minor points

I think the manuscript would be improved by the substitution of the term episode with epoch or session. There two common literatures where this word has a clear meaning; in cognitive science/psychology to refer to episodic memory, and in reinforcement learning to correspond to discrete periods of learning. Neither of these relevant for the results and the use of the term throughout the manuscript was a little distracting. Of course the hippocampus is implicated in episodic memory, but unless your experiment specifically addresses this, then I think it is best left as a discussion point. See Khatib 2023 and Geva et al., 2023 for recent examples with similar experimental designs where other terms were used.

Some of the figures have “Graphic created with BioRender.com” in the legend. This should be moved to the acknowledgements? Or the Methods? The editor should decide, but it shouldn’t go in the figure legends.

“Median difference” in fig1 F (and others). This took me a while to understand what this was, showing the full distribution of the bootstrap estimate of a value is unusual. Perhaps include the term “estimate” the y-axis? Also consider marking the estimated median on the data plot above and having a graphical link of some sort eg dotted line.

## Author Response

We would like to thank the 2 reviewers and the reviewing editor for their time spent reading and thinking about our manuscript, and the helpful feedback they provided us which we believe has led to significant improvement. We revised the manuscript with new analysis, figures, and clarifications to the text that we think address all the reviewer concerns. Our responses to reviewer comments below are in blue and any changes to the manuscript text are shown in red.

Manuscript Instructions

- Extended Data should be labeled as Figure 1-1, Figure 1-2, Table 1-1, etc., so they indicate which figure they are supporting (i.e. Extended Data table supporting Figure 5 labeled as Figure 5-1). This change is not necessary at this stage, but will be if your paper receives a revise decision.

Done.

---------------------------------------------

Synthesis of Reviews:

Synthesis Statement for Author (Required):

The manuscript was reviewed by 2 reviewers and the Reviewing Editor. We agreed that the paper presents a highly interesting re-analysis of previous data. Establishing a relationship between place cell precision and tendency to remap has the potential to explain at least some of the variability in place cell firing.

We greatly appreciate the positive comments on our paper!

Given the potential importance and influence of the interpretation presented in the paper, we feel there is an opportunity to present the data in a more convincing manner, which might involve additional analysis and different presentation of the data. The full reviews are appended below, which provide the complete request, and to which a full response is requested.

In brief, it would be beneficial to convince the reader that the observed effect is present within each/most animal(s), which would counter the critical challenge that the effect only is observed due to systematic differences between individual animals.

We completely agree that this is an important issue to address. We have therefore made a new Extended Data Figure (Fig. 1-3) that shows the Median precision of stable, remapped, and vanished PFs on a mouse-by-mouse basis. As you can see, each mouse shows the same relationship as is shown when all data are pooled together. That is, remapped PFs are less precise than stable and vanished PFs. This shows that our findings are not due to systematic differences between individual animals.

Secondly, a more convincing narrative that the remapping (or otherwise) of place cells is genuinely related to the same cell being recorded over time would be helpful.

We have now added additional details regarding how we determined the same cells were imaged across days. We have also done further analysis to show that the location of place cell somas in the FOV is not related to their fate category (stable, remapped, vanished - Fig. 1-4). These additions are explained in detail in the specific responses to reviewers below.

Reviewer 1

First and foremost, I would like to commend the authors for addressing a crucial aspect of hippocampal place cell dynamics. The study is well-structured, well-quantified and the findings provide valuable insights into the adaptability and flexibility of the hippocampal spatial code. The findings related to the early-stage spatial precision of place fields and their tendency to remap or stabilize, even when excluding effects of backward shifting dynamics, are intriguing.

We thank the reviewer for their encouraging comments.

It might be helpful to perform more population level analysis and simulation to illustrate the potential function of such a dynamic code in memory encoding and retrieval.

We agree that investigating the function of this dynamic code is very important and a direction in which the field should take. There are many ways to investigate this through circuit manipulations and behavioral experiments, as well as computational modeling. However, we believe that the complexities of such an investigation, as well as all the careful controls that would be necessary, makes this line of research beyond the scope of this paper.

Further experiment might be worth trying to solve the causal relationship between the place field precision and remapping tendency.

This is again a very worthy investigation, and we thank the reviewer for bringing it up. Our working model suggests that synaptic plasticity at CA3-CA1 synapses and subsequent homeostatic normalization processes are involved in linking lap-lap PF dynamics with long term stability/remapping/vanishing of PFs (see Discussion section for details). It is of course extremely technically challenging to manipulate synaptic plasticity and homeostatic normalization in vivo during behavior. A computational modeling approach is likely better suited and could reveal important insights. We are currently developing a spiking model of CA1 pyramidal cells with CA3-CA1-like synapses where we can implement plasticity rules to try and match experimental data collected in the lab. However, this modeling approach will take many more months to develop, and a sophisticated exploration of plasticity rules based on STDP and BTSP plus homeostatic normalization processes will extensively expand the scope of the paper. We believe it is better to publish these findings without a computational model and publish a separate modeling paper that thoroughly investigates the synaptic and homeostatic rules that could produce results that match our experimental findings.

Reviewer 2

The authors present reanalysis of data from two recent studies Dong et al., 2021 and Katib et al., 2023, which include place cell recording from dorsal CA1 made using calcium imaging and virtual reality. The authors observe that the precision of a place cell predicts its upcoming fate in a number of different changes to the context. The precision of the place cell refers to the variance of the peak lap-by-lap location; the fate corresponded to a stable, remapped, or vanished place cell in the three changed contexts. This is an intriguing finding and suggests that there are common latent factors for each cell that are reflected in these measures. The authors do a good job at accounting for the backwards shift in place field firing and reporting other characteristics of place fields. Moreover, the authors are to be commended for tackling trial-to-trial variation as a topic of interest since this information often gets lost in averaging.

We thank the reviewer for their accurate summary and interpretation of the data, as well as their positive comments on the paper.

However, despite this, there are a number of concerns I have with the manuscript. The most important of these is confirmation that the effect is real. My other main concern refers to the source of the data, and inconsistencies with the previously reported animal numbers and conditions. If, these points are addressed, I would have much more confidence in the results, and would have no reservations about recommending publication.

These are important concerns, and we believe we have addressed each one below. We would also like to thank the reviewer for their very helpful and insightful suggestions. It is very pleasing to receive such carefully thought-out comments that are clearly designed to make the science and communication of results better. This is of course the purpose of peer-review, but it doesn’t always happen. Thank you.

Animal numbers and data reuse

I cannot seem to make the animal numbers agree with the previously published manuscript (Dong et al., 2021). Where there were 4 mice reported for the full F; F N1; F N2 sequence. There are three different cell counts given for different conditions, and I can’t work out which animals went into which one.

As far as I can gather from the methods in the current manuscript the following is reported

DAY1 DAY2

Mouse# Training F N1 F N2

1 + * *

2 + * *

3 + * * * *

4 + * *

5 + * *

Please fully report the a) behavioural sequence for all animals/groups

This is now reported in a new table (Table 1).

b) the imaging data collected (and if collected and not used, why). A table (such as the one above) and which also includes from which previous publication each animal comes from would probably help future readers.

We apologize for the lack of clarification on this. It is partly because some animals in the Dong et al paper were exposed to a novel environment but not tracked across days, whereas other animals were exposed to N and tracked across days (these are the datasets we used in this paper). Further, not all animals in Dong et al were tracked across 2 days in F. Based on this reviewer’s suggestion we have now added a table to the manuscript that describes exactly which mice were used in which conditions.

Presentation of novel vs familiar results separately

If my interpretation of the which animals were recorded from in each condition is correct (see above), I understand why these data have been split up. You only have one animal for which you have HPC recording for the same cells across all the experimental epochs.

The reviewer does indeed have this correct.

However, the Dong et al., paper suggests you do have this data (at least for the four animals presented there). The authors need to either explain the discrepancy, OR, provide an analysis which considers the fate of each cell for a both a novel and a familiar day1 vs day2. Ie are cells that are unstable in day1F also unstable in day1N1?

I think this might be a misunderstanding because we did not make things clear in the original version. One potential misunderstanding is that N1 and N2 in Dong et al are different environments. Another point is that the across days data analysis in the F condition comes from animals that were exposed to F across 2 days before they were ever exposed to N1 and not from the F exposures in the subsequent sequence of F-N1day1-N1day1-F-N2day1-N2day2. This should be made clear now in the table where we explicitly state which conditions each animal went through.

Precision and remapping are potentially confounded by ROI quality

It is not unreasonable to think that an ROI with poor signal-to-noise will show a more variable PF response within a session due to, for instance, higher contamination from other cells (either neuropil or other nearby soma); in a subsequent session, this cell might be easier to mistake for another cell and so seem to be remapped. The authors’ observation that peak amplitudes of calcium transients (and other parameters) was unchanged across groups would suggest this is not the case, but I think it still needs checking.

I would suggest that the authors look at a metric of ROI isolation quality and see if it is different between the three cases. A quick-and-dirty check for this would be to use the distance from the center of the FOV as a quality metric proxy, since it looks from your imaging that the center has the best imaging. I won’t specify a metric, as they are often included with whatever ROI finding pipeline one uses, so any reasonable one would do.

Excellent suggestion! We did this and made a new figure (fig. 1-4). Because of the difficulty in pooling data across different FOVs, we instead did this analysis on example FOVs (each FOV taken from one mouse) from each of the experiments - one FOV in the F condition, one FOV from the N condition, and one FOV from the R condition. We measured the location of remapped, stable and vanished PCs across the FOVs to check whether there was a correlation between location in the FOV and the fate of the PCs. We found that there is no statistically significant difference in terms of distance from center of the FOV of place cells and the category they belong to (stable, remapped, vanished). This means one population could not be specifically affected by an imaging artifact over the other. All categories would be equally affected.

Also, the results in Fig. 3 were taken during a single imaging session, and so did not require any across-days alignment. The results from that experiment match the results from the across-days experiment; that remapped PFs across epochs are associated with lower lap-by-lap precision in the initial episode. This suggests that aligning FOVs across days is not driving our findings. More details regarding across days alignment are described below and have been added to the paper.

Tracking cells over sessions

I did not feel like the cell tracking over days was sufficiently explained. I tried to find details in Dong et al., 2021 and did not find any. Following the references, it seems that the method for ROI extraction is originally from Mukamel et al., 2009, which describes an activity based, ICA method. Are you running the analysis separately for each day? If so, how do you detect vanished cells in day 2? how do you in general perform correspondence across days? The central concern here is that if you don’t see a cell, it could be silent, or the FOV could have shifted so you are not detecting it.

We agree that these details are missing from the Dong et al paper, and are important for the results presented here. We have now added a more detailed description to the Methods section of how cells were tracked across days. Firstly, we restricted all our analysis to FOVs that were perfectly aligned in the X, Y, and Z planes across days. If we were unable to visually identify the same individual cells by eye, we did not use those FOVs. You can see from the example below, which is also in Fig. 2 of the paper, our FOVs are very accurately matched (day1 on the left and day2 on the right). You can make out blood vessels (black) and individual cells. Aligning FOVs across multiple days is something we do regularly in the lab. To do this we take an average image at the end of the imaging session on day1 and use it as a template to find the exact same FOV the following day in real-time (i.e. before we start collecting data on that day). We match the FOVs within 1um of z-plane alignment (that is the limit of our microscope controller). We further check alignment by concatenating the FOVs across days and motion correcting them together as one single time-series movie. This corrects for any differences in X and Y position and allows us to closely inspect any z-differences at the frame transition from day1 to day2. We do not use any FOVs that have any noticeable differences in z-planes at the transition. All the data shown in this paper are matched in X, Y, and Z.

Place field responses over conditions

The authors do not show enough place cell data in their figures, only showing fluorescent traces in time with position above for a single cell for 2 of three figures. This type of plot of is not suitable to assess the precision of a place cell, which underlies the whole result.

We do show individual place cell examples with stable, remapped, and vanished PFs in figure 1E and 3B. They are not conventional heat map plots, but instead show the location of the transient peaks on each lap (as it is these peaks that we analyze to get a measure of precision).

I would like to see conventional heatmap plats which show the fluorescence for each lap, aligned to the track position (eg Dong et al., fig 2c). I think that showing at least one example from each category (stable, remapped and vanished) would strengthen the presentation of the results.

We have additionally added conventional heatmaps for the examples already shown, to help readers understand the data better (Fig. 1D).

Effect per animal

I would like to see if the effect is true within animal. One concern with this type of result is that it is driven by some individual difference between animals. For instance, one animal just has less precise place cell response, and more remapping overall due to difference in behavior or something about the imaging or the expression. This would drive a measured difference between the cases if all the cells are treated as independent, as they have been in these analyses. The most conservative way to test for this is to perform separate statistical tests for each animal; an alternative would be to use a mixed effects model with the animal ID being a random term; the minimum I would like to see would be at least the effect going in the same direction for each animal, or if that is not the case, reporting it. This is a common issue in systems neuroscience (see Lazic et al., 2010 BMC Neuroscience).

This is an important point, and we agree with the reviewer that this needs to be shown. We have thus added a new figure (Fig. 1-3) that shows the mean values for each animal in regard to precision in the three groups (stable, remapped, vanished). You can see that each mouse closely follows the effects from pooled data. We ran a one-way ANOVA test on the individual mouse plots in this new figure and found all our observations from pooled data held up. We thank the reviewer for this suggestion, which has resulted in a new figure that strengthens our conclusions.

Minor points

I think the manuscript would be improved by the substitution of the term episode with epoch or session. There two common literatures where this word has a clear meaning; in cognitive science/psychology to refer to episodic memory, and in reinforcement learning to correspond to discrete periods of learning. Neither of these relevant for the results and the use of the term throughout the manuscript was a little distracting. Of course the hippocampus is implicated in episodic memory, but unless your experiment specifically addresses this, then I think it is best left as a discussion point. See Khatib 2023 and Geva et al., 2023 for recent examples with similar experimental designs where other terms were used.

This is an interesting point. We tend to agree that avoiding the term “episodic” might avoid confusion about what we mean in this paper. We have therefore changed the term “episode” to “epoch” throughout the paper, including in the title, which now reads:

“The Precision of Place Fields Governs Their Fate Across Epochs of Experience”

Some of the figures have “Graphic created with BioRender.com” in the legend. This should be moved to the acknowledgements? Or the Methods? The editor should decide, but it shouldn’t go in the figure legends.

This has now been removed from the legend and placed in the Methods.

“Median difference” in fig1 F (and others). This took me a while to understand what this was, showing the full distribution of the bootstrap estimate of a value is unusual. Perhaps include the term “estimate” the y-axis?

Added.

Also consider marking the estimated median on the data plot above and having a graphical link of some sort eg dotted line.

The median is actually shown in the data plot, the line next to the data shows the median (the middle blank space) and the interquartile range (the ends of the line).
